# SINFONIA: Scalable Identification of Spatially Variable Genes for Deciphering Spatial Domains

**DOI:** 10.3390/cells12040604

**Published:** 2023-02-13

**Authors:** Rui Jiang, Zhen Li, Yuhang Jia, Siyu Li, Shengquan Chen

**Affiliations:** 1 MOE Key Laboratory of Bioinformatics and Bioinformatics Division, BNRIST/Department of Automation, Tsinghua University, Beijing 100084, China; 2 School of Statistics and Data Science, Nankai University, Tianjin 300071, China; 3 School of Mathematical Sciences and LPMC, Nankai University, Tianjin 300071, China

**Keywords:** spatial transcriptomics, spatial autocorrelation, spatially variable genes, spatial domains

## Abstract

Recent advances in spatial transcriptomics have revolutionized the understanding of tissue organization. The identification of spatially variable genes (SVGs) is an essential step for downstream spatial domain characterization. Although several methods have been proposed for identifying SVGs, inadequate ability to decipher spatial domains, poor efficiency, and insufficient interoperability with existing standard analysis workflows still impede the applications of these methods. Here we propose SINFONIA, a scalable method for identifying spatially variable genes via ensemble strategies. Implemented in Python, SINFONIA can be seamlessly integrated into existing analysis workflows. Using 15 spatial transcriptomic datasets generated with different protocols and with different sizes, dimensions and qualities, we show the advantage of SINFONIA over three baseline methods and two variants via systematic evaluation of spatial clustering, domain resolution, latent representation, spatial visualization, and computational efficiency with 21 quantitative metrics. Additionally, SINFONIA is robust relative to the choice of the number of SVGs. We anticipate SINFONIA will facilitate the analysis of spatial transcriptomics.

## 1. Introduction

Spatially resolved transcriptomics grants us a unique perspective on coherent spatial and gene expression patterns and hence allows for insights into the molecular organization of tissues. Advanced technologies for spatial transcriptomics (STs) have enabled genome-wide profiling of expression levels, demanding scalable methods that take advantage of spatial context to facilitate the identification of spatially variable genes (SVGs), which is regarded as the first critical step in ST data analysis [1]. We note that the task of SVG identification, an essential step before spatial domain characterization, is different from that of spatial pattern visualization and detected-gene evaluation, which are performed after spatial domain characterization, and that the former aims to select individual genes and visualize the expression of the gene in different spatial domains [2,3], while the latter aims to evaluate the differentially expressed genes between different spatial domains [4,5].

Li et al. have provided a survey of computational methods for SVG detection, including eight tools implemented in R and five tools implemented in Python [6]. In recent years, many researchers have preferred to analyze data in Python due to its attractive syntax and highly optimized scientific computing libraries for machine learning [7]. Additionally, in the single-cell community, a number of advanced frameworks have been implemented in Python, such as Scanpy [8], scvi-tools [9] and Squidy [3], suggesting the wide acceptance of Python in single-cell studies. In this study, we focus on Python-based methods and aim to provide an effective and scalable method for the Python community.

Among the tools implemented in Python, SPADE integrates high-level image features and spatial transcriptomes to identify genes that show rich patterns in morphological context [10]. SpatialDE decomposes the variation of each gene into spatial and nonspatial components and identifies SVGs through the comparison between the full model and a model without spatial random effect term [1]. GPcounts is built on a Gaussian process regression model and utilizes a negative binomial likelihood function to model the temporal and spatial variations in gene expression profiles [11]. SOMDE first clusters the neighboring cells into separated nodes based on the self-organizing map (SOM), and then fits the node-level gene expression variation with a Gaussian process to identify SVGs [12]. HMRF uses hidden Markov random fields to identify spatial domains based on cross-platform cell-type mapping [13].

However, these methods are still impeded by relatively low effectiveness and scalability. Additionally, considering that many newly developed methods for STs (e.g., STAGATE [14], stPlus [15], Squidpy [3]) are built on the AnnData format and can be seamlessly integrated into the SCANPY workflow [8], new tools should provide interoperability with AnnData and SCANPY. In addition, the performance of the identified SVGs for deciphering spatial domains still lacks systematic, and especially quantitative, evaluation using the ST datasets with a gold standard.

Spatial autocorrelation statistics, such as Moran’s *I* and Geary’s *C*, have been adopted to select individual genes for spatial pattern visualization (e.g., Seurat [2] and Squidpy [3]), and to quantify the existence of spatial patterns of detected genes (e.g., SpaGE [4] and SpaGCN [5]). However, spatial autocorrelation statistics have not been used to identify SVGs for the characterization of ST data (e.g., dimension reduction) and downstream analyses of ST data (e.g., spatial domain identification). Additionally, the low computational efficiency of existing implementations for calculating the statistics limits their scalability to high-throughput data.

To fill these gaps, we propose a method named SINFONIA to identify spatially variable genes for deciphering spatial domains. Specifically, we construct a spatial neighbor graph and, for the first time, apply spatial autocorrelation statistics to identify SVGs for deciphering spatial domains. We propose an ensemble strategy based on spatial autocorrelation statistics to better identify SVGs and achieve about a four- to two-hundred-and-sixty-two-times speedup compared with other spatial autocorrelation statistics implementations. Additionally, it is the first time that a systematic and quantitative evaluation of SVG identification performance has been conducted using a number of ST datasets.

## 2. Materials and Methods

### 2.1. Construction of Spatial Neighbor Graph

**Figure 1:**
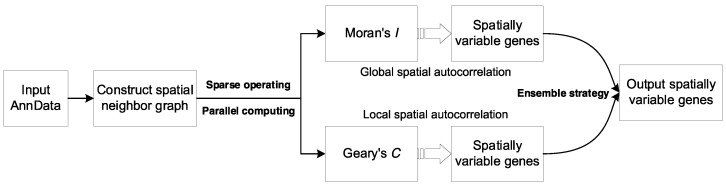
The framework of SINFONIA.

As the SINFONIA framework shown in Figure 1, SINFONIA identifies SVGs based on Moran’s *I* and Geary’s *C* statistics. To calculate the spatial autocorrelation statistics, we construct a spatial neighbor graph (SNG), considering that aggregating information from each spot’s neighbors can improve the characterization of spatial patterns and, consequently, SVGs [16]. Specifically, for each spot we use spatial coordinates to identify k nearest neighbors in a Euclidean space. The weights W of SNG are then defined by (1).

(1) $$\;w_{ij}=\{\begin{array}{l} 1-\frac{D_{ij}}{\max\left(D_{i.}\right)}\;\;\;\;\;if\;spots\;i\;and\;j\;are\;neighbors\\ 0\;\;\;\;\;\;\;\;\;\;\;\;\;\;\;\;\;\;\;\;\;\;\;otherwise\;\;\;\;\;\;\;\;\;\;\;\;\;\;\;\;\;\;\;\;\;\;\;\;\;\;\;\;\;\;\;\;\;\;\;\;\;\;\; \end{array},$$

where $w_{ij}$ denotes the weight between spots i and j, $D_{ij}$ denotes the distance between spots i and j, and $\max\left(D_{i.}\right)$ denotes the maximum distance between spot i and its nearest neighbors. We implemented the calculation via sparse matrix operations, which decrease the space complexity from $O\left(n^{2}\right)$ to O(n) (n≫k), and thus enable memory-efficient and high-speed calculation for the large-scale spatial transcriptomic (ST) data.

### 2.2. Calculation of Spatial Autocorrelation Statistics

We then compute Moran’s *I* and Geary’s *C* for each gene based on W, the former of which characterize spatial autocorrelation in the given gene among neighbors in the SNG. In ST data, the gene expression in spots from localized spatial neighborhoods tends to be closer in value than those at distant locations. For example, the expression levels of a gene at nearby locations may be closer in value than expression levels at distant locations. Therefore, genes with stronger spatial autocorrelation can exhibit more organized spatial expression patterns. Here, we calculate spatial autocorrelation statistics based on our constructed SNG to quantify the spatial autocorrelation degree of each gene.

Specifically, Moran’s *I* measures the overall spatial autocorrelation of a dataset [17]. Given a gene and SNG of the spots, Moran’s *I* evaluates whether the spatial expression pattern is clustered, dispersed, or random. The Moran’s *I* score ranges from −1 to 1, where a score close to 1 indicates a clear spatial pattern, a score close to 0 indicates random spatial expression, and a score close to −1 indicates a chess board-like pattern. For each gene, the Moran’s *I* score is computed as (2):

(2) $$\;\;\;I=\frac{N}{W}\frac{\sum_{i}\sum_{j}\left[w_{ij}\left(x_{i}-\bar{x}\right)\left(x_{j}-\bar{x}\right)\right]}{\sum_{i}{\left(x_{i}-\bar{x}\right)}^{2}},$$

where $x_{i}$ and $x_{j}$ denote the gene expression levels of spots i and j, respectively, $\bar{x}$ is the mean expression level of the gene, N is the total number of spots, $w_{ij}$ is the edge weight between spots i and j in SNG, and W is the sum of SNG weights.

Geary’s *C*, another commonly used statistic [18], is inversely related to Moran’s *I*, but is not identical to Moran’s *I*. Compared with Moran’s *I*, which measures global spatial autocorrelation, Geary’s *C* is more sensitive to local spatial autocorrelation. For each gene, the Geary’s *C* score is computed as (3):

(3) $$\;\;\;\;\;\;\;C=\frac{\left(N-1\right)\sum_{i}\sum_{j}\left[w_{ij}{\left(x_{i}-x_{j}\right)}^{2}\right]}{2W\sum_{i}{\left(x_{i}-\bar{x}\right)}^{2}}$$

The score of Geary’s *C* ranges from 0 to 2. To make it on the same scale as that of Moran’s *I*, we rescale it by (4):

(4) $$\;\;C^{*}=1-C,$$

where a $C^{*}$ close to 1 means positive spatial autocorrelation, a $C^{*}$ close to 0 means poor spatial autocorrelation, and a $C^{*}$ close to −1 means negative spatial autocorrelation.

### 2.3. Identification of Spatially Variable Genes

SINFONIA identifies SVGs for spatial domain detection based on an ensemble strategy for Moran’s *I* and Geary’s *C*. Given a specified number n of SVGs, SINFONIA_*I*, a variant of SINFONIA, selects the top n genes with the highest Moran’s *I* scores as SVGs, while SINFONIA_*C*, another variant of SINFONIA, selects the top n genes with the highest rescaled Geary’s *C* scores as SVGs. In this work, following the SCANPY workflow for ST data, we identified the top 2000 SVGs with SINFONIA_*I*, SINFONIA_*C* and other baseline methods by default on all datasets. SINFONIA adopts the union of SVGs identified by SINFONIA_*I* and SINFONIA_*C* as SVGs, based on an ensemble idea that integrates both global and local spatial autocorrelation. The ensemble strategy provides overall better performance than the two variants and is more robust than the variants, which fluctuate using different downstream clustering methods.

### 2.4. Implementation and Usage of SINFONIA

With the increase of data throughput and data scale, computational methods should meet the critical principles of efficiency and scalability [14]. To achieve superior efficiency and scalability, we implemented the calculation of Moran’s *I* and Geary’s *C* via sparse matrix operations and implemented parallel computing for genes. Specifically, SINFONIA performs sparse matrix operations using SciPy 2-D sparse array grammar for numeric data [19], which focuses on the calculation of only non-zero values, and thus enables memory-efficient and high-speed calculation for large-scale data. Additionally, SINFONIA adopts Numba (https://numba.pydata.org/ accessed on 17 July 2022) to translate functions to optimized fast machine code and achieve parallel computing. In addition, with tailored programming, SINFONIA computes Moran’s *I* and Geary’s *C* simultaneously with almost no extra time consumption compared to computing a single statistic.

The AnnData format offers a convenient way to store data matrices and annotations together. The use of AnnData format also facilitates interoperability with existing single-cell analysis tools such as SCANPY [8]. We designed the structure of the SINFONIA class to smoothly interface with the widely-used AnnData format. Therefore, SINFONIA can be seamlessly integrated into the SCANPY vignette by taking the AnnData object as input, and replace the default SVG identification method via just a single line of code. In addition, its modularity makes it efficient for data representation and storage, and makes it flexible to be interfaced with various external tools in the Python data science and machine-learning ecosystem, such as advanced deep-learning frameworks. Nevertheless, existing SVG identification methods in the Python community, such as SpatialDE [1] and SOMDE [12], have poor interoperability with the widely-used AnnData format and SCANPY workflow, and thus hamper the usage and application of these methods.

Additionally, SINFONIA provides rich documentation in the form of functional application programming interface documentation, tutorials and example workflows. It is easy to navigate and is accessible to both experienced developers and beginner analysts.

### 2.5. Data Collection

We systematically assessed the performance of SINFONIA using 15 ST datasets generated with different protocols, and with different sizes, dimensions and qualities. We note that limited sequencing coverage of single-cell technologies, especially for STs, results in large fractions of observed zeros, namely a high degree of data sparsity, providing data with low quality [20].

First, we collected a 10X Genomics Visium dataset containing spatial expressions of 12 human dorsolateral prefrontal cortex (DLPFC) sections from spatialLIBD [21]. The spots were manually annotated based on morphological features and gene markers [22]. 

Second, we downloaded 10X Genomics Visium ST data of a mouse brain coronal (MBC) section [23]. The annotation was collected from Squidpy [3], which annotated the spots using several resources, including the Allen Brain Atlas [24], the Mouse Brain gene expression atlas (http://mousebrain.org/ accessed on 17 July 2022), and this study [25].

Third, we collected a Slide-seqV2 dataset with 10 μm spatial resolution profiled from mouse hippocampus [26]. This dataset does not come with annotations that can serve as ground-truth since spatial domain detection was not performed in the original study. To evaluate the performance of spatial domain characterization visually, we also retrieved the annotation of hippocampus structures from the Allen Brain Atlas [24].

Fourth, we downloaded a mouse olfactory bulb ST dataset generated by Stereo-seq [27]. Analogously, because of a lack of ground-truth domain annotation for this dataset, we relied on the Allen Brain Atlas [24] to evaluate the consistency between the identified clusters and the annotated tissue structures.

A summary of the fifteen ST datasets used for performance evaluation is provided in Table 1.

### 2.6. Performance Evaluation

Deciphering spatial domains (i.e., regions with similar spatial expression patterns) is one of the great challenges for STs [14]. We illustrate the benefits of SINFONIA for deciphering spatial domains from five perspectives: spatial clustering, domain resolution, latent representation, spot visualization, and computational efficiency. We identified SVGs by various methods and performed data analysis following the SCANPY vignette for ST data. All the experiments were performed on a standard desktop with an AMD Ryzen 7 1700X Eight-Core CPU with 32GB of RAM.

#### 2.6.1. Spatial Clustering

We performed clustering on the latent representation of spots obtained from principal component analysis on expression levels of the identified SVGs following the SCANPY vignette for ST data [8]. Two widely-used community detection-based clustering methods, i.e., Louvain clustering and Leiden clustering, with default resolution in SCANPY, were adopted. In addition, for the situation where the number of clusters is specified, we implemented a binary search to tune the resolution parameter in clustering to make the number of clusters and the specified number as close as possible. The number was specified as the unique number of ground-truth labels in our benchmark experiments. For the binary search strategy, we searched the resolution in the range from 0.0 to 3.0. If the number of clusters did not match the specified number, the resolution value inducing the closest number of clusters to the specified number was used to perform clustering in the next iteration.

We considered the first scenario since the number of ground-truth spatial domains is unknown in advance in most studies and Louvain or Leiden clustering with default resolution is usually adopted [8,28]. The benchmark results in this scenario can indicate the performance of different SVG identification methods in general ST applications. We also considered the second scenario, one inspired by several studies for benchmarking single-cell analysis performance [15,29,30,31,32]. The benchmark results in this scenario can indicate the performance of different methods more fairly and objectively because the number of clusters is made to be as close as possible to the number of spatial domains. Briefly, the former is more general and can be considered as an evaluation of real data, while the latter is more specific and can be considered as an evaluation of simulation data.

We evaluated the clustering results based on four widely-used metrics: adjusted mutual information (AMI) [33], adjusted Rand index (ARI) [34], homogeneity (Homo) [35], and normalized mutual information (NMI) [36]. Note that AMI is preferred for unbalanced clusters, while ARI is more suitable for ST datasets with large balanced clusters [15,32,37]. Since the sizes of spot populations in most ST data, including the datasets used in this study, are unbalanced, AMI is more appropriate than ARI in the benchmarking results.

To be more specific, let T denote the ground-truth labels of spots, P denote the clustering results, N denote the total number of spots, $x_{i}$ denote the number of spots assigned to the i-th unique cluster of *P*, $y_{j}$ denote the number of spots that belong to the j-th unique label of T, $n_{ij}$ denote the number of overlapping spots between the i-th cluster and the j-th unique label, MI(·,·) denote the mutual entropy, E(·) denote the expectation function, H(·) denote the entropy function, and H(T|P) denote the uncertainty of ground-truth labels based on the knowledge of clustering results. Then the AMI score is calculated as (5):

(5) $$\;\mathrm{AMI}=\frac{MI\left(P,T\right)-E\left[MI\left(P,T\right)\right]}{avg\left[H\left(P\right),H\left(T\right)]-E[MI\left(P,T\right)\right]}$$

The ARI score is computed as (6):

(6) $$\;\;\;\mathrm{ARI}=\frac{\sum_{ij}\left(\begin{array}{c} n_{ij}\\ 2 \end{array}\right)-\left[\sum_{i}\left(\begin{array}{c} x_{i}\\ 2 \end{array}\right)\sum_{j}\left(\begin{array}{c} y_{j}\\ 2 \end{array}\right)\right]/\left(\begin{array}{c} N\\ 2 \end{array}\right)}{\frac{1}{2}\left[\sum_{i}\left(\begin{array}{c} x_{i}\\ 2 \end{array}\right)+\sum_{j}\left(\begin{array}{c} y_{j}\\ 2 \end{array}\right)\right]-\left[\sum_{i}\left(\begin{array}{c} x_{i}\\ 2 \end{array}\right)\sum_{j}\left(\begin{array}{c} y_{j}\\ 2 \end{array}\right)\right]/\left(\begin{array}{c} N\\ 2 \end{array}\right)}$$

The Homo score is calculated as (7):

(7) $$\mathrm{Homo}=1-\frac{H(T|P)}{H\left(T\right)}$$

The NMI score is computed as (8):

(8) $$\mathrm{NMI}=\frac{MI\left(P,T\right)}{\sqrt{H\left(P\right)H\left(T\right)}}$$

#### 2.6.2. Domain Resolution

Intuitively, based on the ground-truth domain labels and the spot embeddings learned from the identified SVGs, discovery of more nearest neighbors with the same domain label indicates better domain resolution and, consequently, better performance for the characterization of spatial domains. As suggested by [38], we evaluated domain resolution by mean average precision (MAP). Supposing that the domain label of the i-th spot is $y^{\left(i\right)}$, and the domain labels of its K ordered nearest neighbors are $y_{1}^{\left(i\right)},{\;y}_{2}^{\left(i\right)},\;\ldots ,{\;y}_{K}^{\left(i\right)}$, the MAP score is computed as (9) and (10).

(9) $$\;\;\mathrm{MAP}=\frac{1}{N}\overset{N}{\underset{i=1}{\sum }}{\mathrm{AP}}^{\left(i\right)}$$

(10) $$\;{\mathrm{AP}}^{\left(i\right)}=\{\begin{array}{c} \frac{\sum_{k=1}^{K}1_{y^{\left(i\right)}=y_{k}}\cdot \frac{\sum_{j=1}^{k}1_{y^{\left(i\right)}=y_{j}^{\left(i\right)}}}{k}}{\sum_{k=1}^{K}1_{y^{\left(i\right)}=y_{k}^{\left(i\right)}}},\;\;if\;\overset{K}{\underset{k=1}{\sum }}1_{y^{\left(i\right)}=y_{k}^{\left(i\right)}}>0\\ 0,\;\;\;\;\;\;\;\;\;\;\;\;\;\;\;\;\;\;otherwise\; \end{array}$$

where $1_{y^{\left(i\right)}=y_{k}^{\left(i\right)}}$ is an indicator function that equals 1 if $y^{\left(i\right)}=y_{k}^{\left(i\right)}$ and 0 otherwise, and K is set to 30. For each spot, average precision (AP) measures the average label precision up to each domain-matched neighbor. MAP, ranging from 0 to 1, is the average AP across all spots, and a higher MAP indicates better domain resolution.

#### 2.6.3. Latent Representation

We also investigated the representation performance of latent embeddings learned from the identified SVGs. First, we directly evaluated the information contained in the latent representation for predicting the true spatial domains in a supervised manner. Specifically, we treated the latent representation as the input and treated the true spatial domains as the output. As suggested by two benchmark studies on supervised cell type identification for single-cell RNA-seq data [39,40], we adopted the support vector machine (SVM) with radial basis function kernel as the classifier. We conducted five-fold cross-validation experiments by randomly splitting all spots into five folds and iteratively predicting spatial domains of the spots in each fold using the model trained with the remaining four folds. We computed the mean accuracy of the five folds (mean cross-validation accuracy, MCVA) to evaluate the predictive ability of the latent representation in predicting the spatial domains. A higher MCVA score suggests that the latent representation learned from the identified SVGs can better extract information in accurately predicting spatial domains.

We further adopted the local inverse Simpson’s index (LISI), a commonly-used integration quality quantification metric in single-cell RNA-seq [41,42], to evaluate the characterized spatial domain patterns in latent representation for each spot. We built Gaussian kernel-based distributions of neighborhoods in the latent space, and calculated the LISI score using the compute_lisi function from the LISI R package with default parameters (perplexity = 30). A lower LISI score means more homogeneous neighborhood spatial domains of the spot [41,42]. To facilitate the benchmark, we used the transformed LISI scores, namely the tinverse of the median or mean LISI (iLISImd or iLISIm), to evaluate the performance. A higher iLISImd or iLISIm score suggests that the latent representation contains more effective information for deciphering spatial domains.

#### 2.6.4. Spot Visualization

With the spatial coordinates and clustering results of spots, we assessed the performance of SVG identification methods via spot visualization. We collected annotations of related tissues from the Allen Reference Atlas [24], and used the annotation as a reference. We can evaluate the performance by visually investigating whether different domains were clearly segregated, whether the detected domains were spatially continuous and smooth, whether the revealed spatial domains were well consistent with the collected annotations, and whether the known tissue structures were successfully identified.

#### 2.6.5. Computational Efficiency

Advanced high-throughput technologies for spatial transcriptomics have generated massive ST datasets, impeding exploratory data analysis on standard desktops. Therefore, we also benchmarked the computation time of various methods. We first compared the computation time of SINFONIA and its two variants with other SVG identification methods, including hvg [8], SOMDE [12], and SpatialDE [1]. Second, we compared the computation time of SINFONIA and its two variants with other Python implementations for the calculation of Moran’s *I* and Geary’s *C*, including Squidpy [3] and SpaGCN [5]. We note that the implementation in SpaGE [4] is the same as that in SpaGCN.

To sum up, we evaluated SVG identification performance by twenty-one metrics, including MAP, MCVA, iLISImd, iLISIm, computation time, and four clustering metrics for two clustering methods with default resolution or specified cluster number.

### 2.7. Baseline Methods

We compared the performance of SINFONIA with three baseline methods, including SpatialDE [1], SOMDE [12], and hvg [8], which is adopted in the widely-used SCANPY workflow for ST data. Specifically, SpatialDE and SOMDE were executed using the Python packages SpatialDE (v1.1.3, https://github.com/Teichlab/SpatialDE accessed on 17 July 2022) and somde (v0.1.8, https://github.com/XuegongLab/somde accessed on 17 July 2022), respectively, while hvg was executed using the SCANPY (v1.9.1, https://github.com/scverse/scanpy accessed on 17 July 2022) implementation of the Seurat method for hvg filtering. We identified SVGs by various methods with the default parameters or settings provided in the accompanying examples. Following the SCANPY vignette for ST data, the top 2000 SVGs identified by various methods were used for benchmarking. We used the standard SCANPY workflow for ST data with default settings to evaluate the performance for characterizing spatial domains.

We also tried to benchmark the performance of other three Python-based methods, including SPADE [10], GPcounts [11], and HMRF [13]. However, SPADE requires additional high-resolution tissue images as input, which are not available on most of the ST datasets. Additionally, it would have led to an unfair comparison, even though the images are available. We then ran GPcounts following its tutorials. However, when we performed GPcounts on the smallest dataset, i.e., the MBC dataset, the status bar demonstrated that the computational time of the method would be more than 1668 h, making it impossible to benchmark the performance of GPcounts. We also tried to benchmark HMRF. Nevertheless, we failed to access the instructions of HMRF (http://spatial.rc.fas.harvard.edu/install.html accessed on 17 July 2022). Therefore, we finally adopted SpatialDE [1], SOMDE [12], and hvg [8] as baseline methods.

## 3. Results

### 3.1. SINFONIA Enables Accurate Spatial Clustering

**Figure 2:**
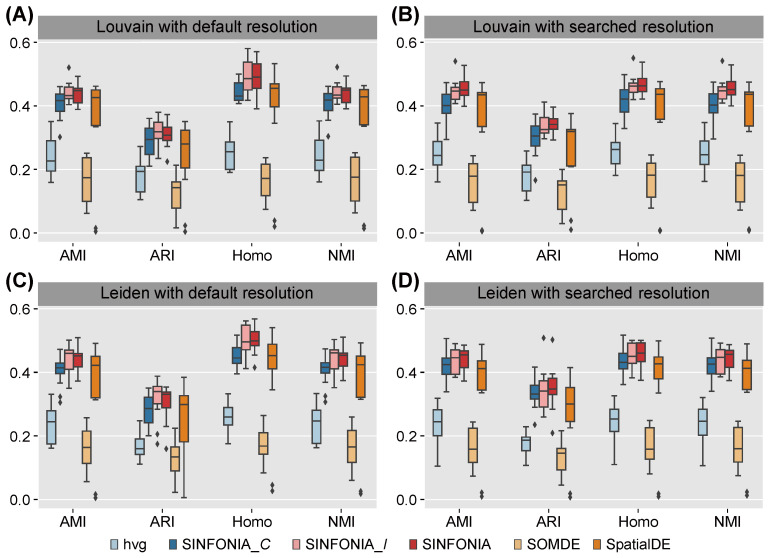
Louvain clustering results with (**A**) default resolution and (**B**) searched resolution (given a specified number of clusters) on 12 DLPFC datasets. Leiden clustering results with (**C**) default resolution and (**D**) searched resolution on 12 DLPFC datasets.

It is intuitive that, based on the ground-truth domain labels and expression levels of the identified SVGs, a higher score of clustering metric indicates better performance for deciphering spatial domains. We first quantitatively evaluated the spatial clustering performance of SINFONIA. To mimic the scenario where the number of spatial domains is unknown, we used Louvain clustering with default resolution. This is a general scenario, since the number of ground-truth spatial domains is unknown in advance in most studies. We performed spatial clustering on each of the 12 DLPFC datasets. As shown in Figure 2A, SINFONIA achieved significantly higher AMI, ARI, Homo and NMI values than did the baseline methods. SpatialDE also achieved satisfactory performance, while with high variance across the 12 DLPFC datasets. Additionally, we considered the scenario where a specified number of clusters is given. The scenario is often adopted in benchmarking studies [15,29,30,31,32]. We used a binary search strategy to obtain clusters, and SINFONIA again outperformed the baseline methods (Figure 2B). In addition, we also evaluated the performance of two SINFONIA variants, namely SINFONIA_*I* and SINFONIA_*C*, to demonstrate the advantages of the ensemble strategy in SINFONIA. The evaluation of SINFONIA and its two variants is equivalent to ablation experiments. As shown in Figure 2A,B, SINFONIA achieved better performance than SINFONIA_*I*, and SINFONIA_*I* achieved better performance than SINFONIA_*C*, indicating that SINFONIA integrates the advantages of Moran’s *I* and Geary’s *C* and thus achieves the best performance. In addition to Louvain clustering, we also assessed the spatial clustering performance of SVGs identified by various methods via Leiden clustering, which is another widely-used clustering method in the single-cell community [2,8]. As shown in Figure 2C,D, Leiden clustering with default resolution or searched resolution also provided similar results, suggesting that the evaluation results are robust relative to clustering methods and SINFONIA consistently outperformed the baseline methods regardless of clustering methods and scenarios.

**Figure 3:**
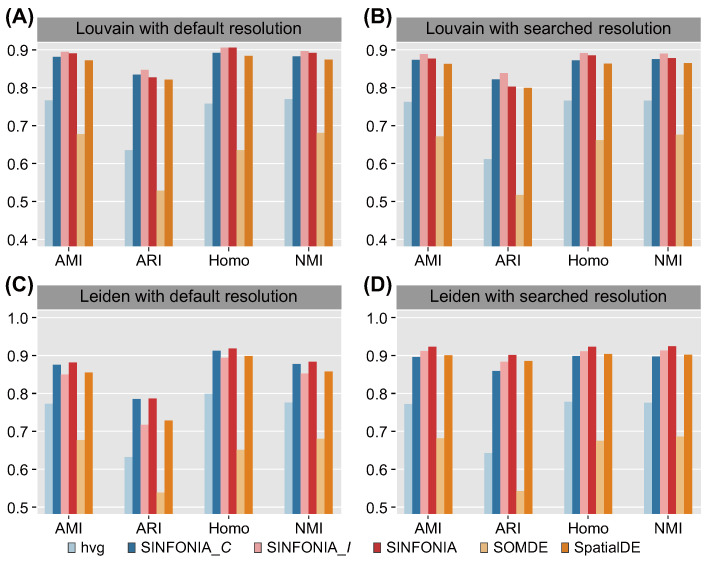
Louvain clustering results with (**A**) default resolution and (**B**) searched resolution on the MBC dataset. Leiden clustering results with (**C**) default resolution and (**D**) searched resolution on the MBC dataset.

To demonstrate the superiority of spatial clustering results across multiple datasets, we further benchmarked SINFONIA on the MBC dataset. As shown in Figure 3A–D, SINFONIA again deciphered the spatial domains better than baseline methods. Additionally, SINFONIA provided overall better performance than its two variants and was more robust than the variants. We note that SINFONIA_*C* outperformed SINFONIA_*I* when clustering by Leiden with default resolution on this dataset, while SINFONIA_*I* outperformed SINFONIA_*C* in other cases and on the 12 DLPFC datasets, suggesting that SVG identification based on an individual statistic may provide fluctuating performance while SINFONIA can provide the overall best performance based on its ensemble strategy.

### 3.2. SINFONIA Effectively Characterizes Spatial Patterns

**Figure 4:**
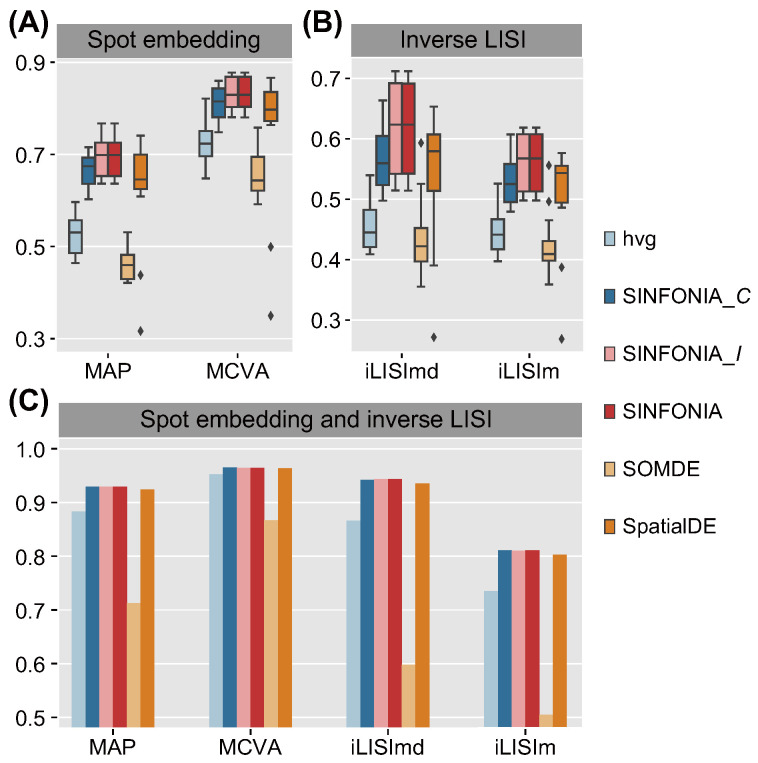
(**A**) Performance of MAP and MCVA on the 12 DLPFC datasets. (**B**) Performance of iLISImd and iLISIm on the 12 DLPFC datasets. (**C**) Performance of MAP, MCVA, iLISImd and iLISIm on the MBC dataset.

To further investigate the effectiveness of spatial patterns that were characterized based on the SVGs identified by the different methods, we assessed the domain resolution by MAP, and assessed the prediction ability by MCVA, iLISImd and iLISIm. Again, SINFONIA achieved consistent superiority over baseline methods and, overall, performed better than its two variants on 12 DLPFC datasets (Figure 4A,B) and the MBC dataset (Figure 4C). Note that hvg, the SVG identification method used in the SCANPY vignette tailored for ST data analysis, provided relatively undesirable performance, which is consistent with the evaluation results in spatial clustering. The above results indicate that a simple replacement of the SVG identification step with SINFONIA can significantly improve the domain resolution and prediction ability of characterized spatial patterns.

### 3.3. SINFONIA Facilitates Interpretable Spot Visualization

Interpretable visualization of spatial domains is important for researchers to better understand tissue structures and study biological functions. We next applied SINFONIA to a mouse hippocampus dataset sequenced by Slide-seqV2 [26] and a mouse olfactory bulb dataset sequenced by Stereo-seq [27]. Since these two datasets lack manually annotated spatial domain labels, we evaluated the interpretability by comparing the spatial domains obtained by clustering (Louvain with default resolution) with the annotated function structures in the Allen Brain Atlas [24].

**Figure 5:**
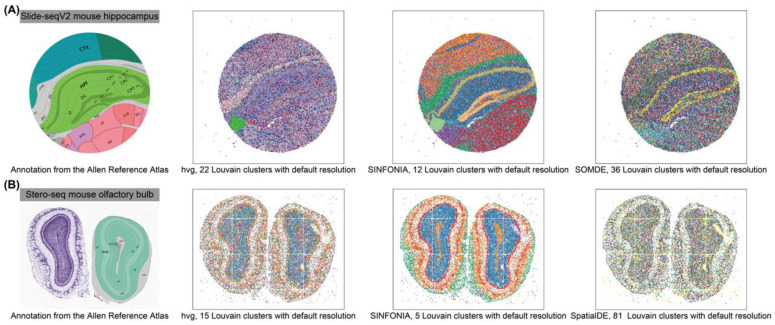
Visualization of datasets of (**A**) a mouse hippocampus and (**B**) a mouse olfactory bulb with tissue regions annotated from the Allen Brain Atlas and spatial domains detected by different methods.

We visualized the spots with the spatial coordinates and clustering results. As shown in Figure 5A,B, on both of these two datasets, the spatial domains revealed based on the SVGs identified by SINFONIA were well consistent with the annotation from the Allen Reference Atlas, while the clusters identified by other methods lacked clear spatial separation, indicating the advantage of SINFONIA for characterizing and visualizing spatial domains. For example, based on the SVGs identified by SINFONIA, we successfully detected the third ventricle (V3), dentate gyrus-granule cell layer (DG-sg), and CA field of pyramidal layer in the mouse hippocampus, and detected the subependymal zone (SEZ) in the mouse olfactory bulb. However, based on the SVGs identified by other methods, most of the tissue structures can hardly be characterized. Additionally, the baseline methods tended to claim significantly more spatial domains than did SINFONIA, which may be due to the limited spatial patterns and the even noise in the identified SVGs. In addition, we note that errors were encountered when performing SpatialDE and SOMDE on these two datasets, respectively, highlighting the importance of user-friendliness and software quality to the success of bioinformatics tools [43].

### 3.4. SINFONIA Is Robust and Computationally Efficient

**Figure 6:**
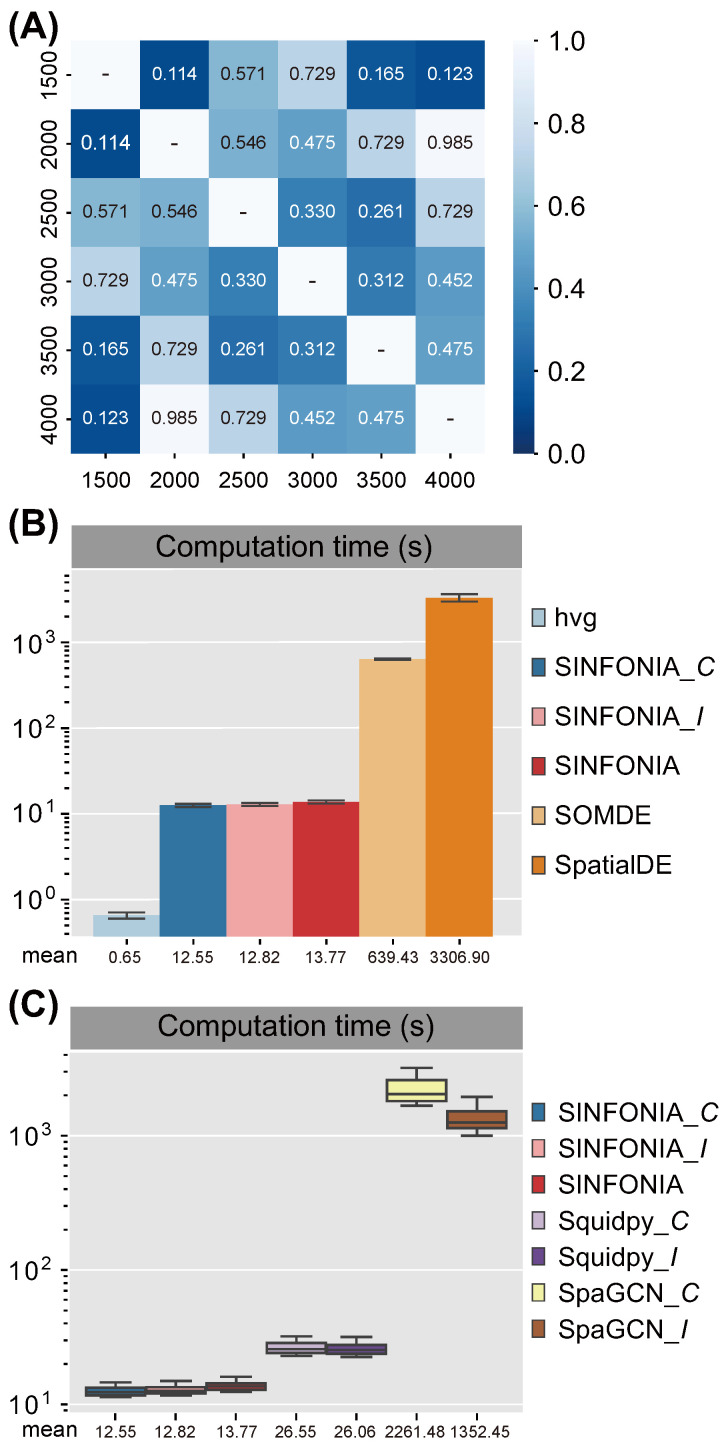
(**A**) *p*-values of two-sided Wilcoxon signed-rank tests on the performance of different numbers of SVGs identified by SINFONIA. (**B**) Computation time of different methods on 12 DLPFC datasets. (**C**) Computation time of different implementations for calculating spatial autocorrelation statistics on 12 DLPFC datasets.

The robustness of hyperparameters is an important aspect that affects the ease of use of a method. The major hyperparameter of SVG identification is the number of SVGs to be selected. In order to investigate the robustness of SINFONIA relative to the choice of the number of SVGs, we further performed SINFONIA on the 12 DLPFC datasets to select different numbers of SVGs. We adopted 20 metrics (i.e., four clustering metrics for two clustering methods with default resolution or specified cluster number: MAP, MCVA, iLISImd, and iLISIm) to demonstrate the robustness. For each choice of the number of SVGs, we computed the median of each metric on the 12 datasets, and thus obtained 20 median metrics. We then performed two-sided Wilcoxon signed-rank tests on different pairs of choice of the number of SVGs to test if the performance has a significant difference using different numbers of SVGs. As shown in Figure 6A, the performance of SINFONIA did not differ significantly using various numbers of SVGs (all the *p*-values were greater than 0.1), indicating that SINFONIA was robust to the choice of the number of SVGs.

To catch up with the growth in data throughput, computational methods should be designed with scalability in mind. Therefore, in addition to the comparison of spatial characterization performance of different SVG identification methods, we also compared the computational efficiency of different methods on the 12 DLPFC datasets. As shown in Figure 6B, SINFONIA and its two variants significantly outperformed the other methods except for the conventional hvg method. Additionally, we also systematically benchmarked the computational efficiency of different implementations for calculating Moran’s *I* and Geary’s *C*. In the Python community of ST data analysis, Squidpy [3] and SpaGCN [5] provide implementations for the calculation of Moran’s *I* and Geary’s *C*. The Moran’s *I* implementation of SpaGCN is the same as that of SpaGE [4]. Hence, we compared the computational efficiency of SINFONIA and its two variants with Squidpy and SpaGCN. We used various implementations to calculate spatial autocorrelation statistics based on the same spatial neighbor graph to ensure the fairness of assessment. As shown in Figure 6C, SINFONIA offered about four- to two-hundred-and-sixty-two- times speedup compared with other implementations for calculating spatial autocorrelation statistics Moran’s *I* and Geary’s *C*. In addition, SINFONIA computed Moran’s *I* and Geary’s *C* simultaneously with almost no extra time consumption compared to computing a single statistic. The above results highlight that the contribution of SINFONIA to the ST data analysis community lies in not only the superior spatial characterization performance, but also the advanced implementation of spatial autocorrelation statistics.

### 3.5. SINFONIA Improves the Performance of Other Spatial Embedding Methods

**Figure 7:**
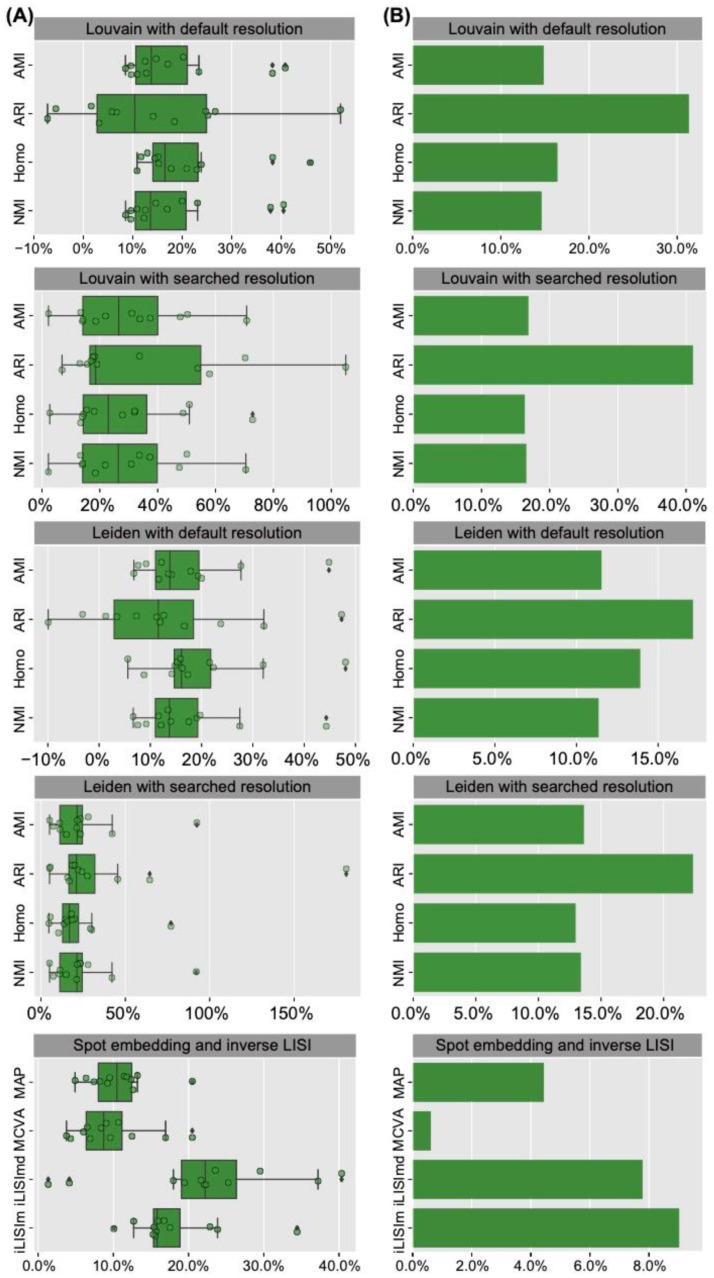
Performance improvement of STAGATE using SINFONIA on (**A**) the 12 DLPFC datasets and (**B**) the MBC dataset. The performance of spatial clustering, domain resolution and prediction ability were evaluated.

In addition to the principal component analysis used in the SCANPY vignette tailored for ST data analysis, we further adopted STAGATE, the state-of-the-art method for ST data embedding [14], to demonstrate that using the SVGs identified by SINFONIA can also improve the performance of more advanced spatial embedding methods. We systematically evaluated the performance of STAGATE on the 12 DLPFC datasets and the MBC section dataset using the SVGs identified by hvg and SINFONIA, respectively. We assessed the performance with spatial clustering, domain resolution and prediction ability. As shown in Figure 7A,B, by simply replacing the SVG identification method from hvg used in SCANPY workflow to SINFONIA, STAGATE can achieve significant improvements. The results of the combination of SINFONIA and STAGATE indicate that our method can be used as a general step in ST data analysis to enhance the performance of existing ST data embedding methods.

## 4. Discussion

Rapid advances in spatially resolved transcriptomics have revolutionized the interrogation of spatial heterogeneity and granted us a novel perspective on the cellular transcriptome. The identification of spatially variable genes (SVGs) is an essential step for downstream spatial domain characterization, and is regarded as the first critical step in spatial transcriptomic (ST) data analysis [1]. In this study, we propose SINFONIA, a scalable method for identifying spatially variable genes via ensemble strategies. We systematically benchmarked the performance of SINFONIA on 15 ST datasets generated with different protocols, and with different sizes, dimensions and qualities. We have demonstrated the applications of SINFONIA from five perspectives, including spatial clustering, spatial domain annotation, spot visualization, spatial embedding and applicability to various ST datasets. Furthermore, we have also shown that SINFONIA is robust relative to the number of SVGs and is computationally efficient. As an ensemble-based method, SINFONIA can provide the overall best performance based on both the global autocorrelation (Moran’s *I*) and the local spatial autocorrelation (Geary’s *C*), highlighting the contribution of the ensemble strategy in SINFONIA for SVG identification. Certainly, although SINFONIA successfully integrates the spatial coordinates and gene expression profiles in ST data to identify SVGs, its performance can be further improved in the future. For example, more advanced computational technologies and hardware such as the graphics processing unit (GPU) can be used to further accelerate the computation of spatial autocorrelation statistics, and thus facilitate the analysis of large-scale ST data.

## 5. Conclusions

This study is the first attempt to apply spatial autocorrelation statistics to the identification of SVGs for deciphering spatial domains. Additionally, it is the first time that a systematic and quantitative evaluation of SVG identification performance has been conducted using a number of ST datasets. SINFONIA, the ensemble-based method proposed in this study, provides an effective way to identify SVGs to facilitate ST data analysis, and achieves about four- to two-hundred-sixty-two-times speedup compared with other implementations. With its smooth interfaces to SCANPY and the Python data science ecosystem, we anticipate broad application of SINFONIA within ST data analysis.

## Figures and Tables

**Table 1:** Summary of the fifteen benchmarking datasets.

Dataset	# of Spots	# of Genes	# of Domains	Sparsity	Protocol	Species	Reference
DLPFC_151507	4221	33,538	7	0.958	10X Visium	*Homo sapiens*	[22]
DLPFC_151508	4381	33,538	7	0.964	10X Visium	*Homo sapiens*	[22]
DLPFC_151509	4788	33,538	7	0.957	10X Visium	*Homo sapiens*	[22]
DLPFC_151510	4595	33,538	7	0.959	10X Visium	*Homo sapiens*	[22]
DLPFC_151669	3636	33,538	5	0.946	10X Visium	*Homo sapiens*	[22]
DLPFC_151670	3484	33,538	5	0.950	10X Visium	*Homo sapiens*	[22]
DLPFC_151671	4093	33,538	5	0.945	10X Visium	*Homo sapiens*	[22]
DLPFC_151672	3888	33,538	5	0.947	10X Visium	*Homo sapiens*	[22]
DLPFC_151673	3611	33,538	7	0.934	10X Visium	*Homo sapiens*	[22]
DLPFC_151674	3635	33,538	7	0.920	10X Visium	*Homo sapiens*	[22]
DLPFC_151675	3566	33,538	7	0.946	10X Visium	*Homo sapiens*	[22]
DLPFC_151676	3431	33,538	7	0.942	10X Visium	*Homo sapiens*	[22]
Brain coronal	2800	32,285	15	0.870	10X Visium	*Mus musculus*	[23]
Hippocampus	53,208	23,264	-	0.982	Slide-seqV2	*Mus musculus*	[26]
Olfactory bulb	19,527	27,106	-	0.987	Stereo-seq	*Mus musculus*	[27]

## Data Availability

The 10X Genomics Visium dataset of 12 DLPFC sections [22] can be downloaded from spatialLIBD at http://spatial.libd.org/spatialLIBD/ (accessed on 17 July 2022) [21]. The 10X Genomics Visium dataset of a coronal mouse brain section is available at https://support.10xgenomics.com/spatial-gene-expression/datasets (accessed on 17 July 2022) [23]. The Slide-seqV2 dataset of mouse hippocampus is accessible at https://singlecell.broadinstitute.org/single_cell/study/SCP815/highly-sensitive-spatial-transcriptomics-at-near-cellular-resolution-with-slide-seqv2#study-summary (accessed on 17 July 2022) [26]. The Stereo-seq dataset of mouse olfactory bulb tissues can be downloaded from https://db.cngb.org/search/project/CNP0001543/ (accessed on 17 July 2022) [27]. The annotated function structures in Allen Brain Atlas are available at https://atlas.brain-map.org/ (accessed on 17 July 2022) [24]. SINFONIA is freely accessible at https://sinfonia-svg.readthedocs.io/ (accessed on 17 July 2022) under the MIT License, along with detailed tutorials and examples. It can be seamlessly integrated into existing analysis workflows. The source code is available at https://github.com/BioX-NKU/SINFONIA (accessed on 17 July 2022).
